# Convergence of Relative Entropy for Euler–Maruyama Scheme to Stochastic Differential Equations with Additive Noise

**DOI:** 10.3390/e26030232

**Published:** 2024-03-06

**Authors:** Yuan Yu

**Affiliations:** School of Statistics and Mathematics, Shandong University of Finance and Economics, Jinan 250014, China; yuyuan_mail@126.com

**Keywords:** relative entropy, Euler–Maruyama scheme, Girsanov’s transform, Hölder continuity, weighted variation distance

## Abstract

For a family of stochastic differential equations driven by additive Gaussian noise, we study the asymptotic behaviors of its corresponding Euler–Maruyama scheme by deriving its convergence rate in terms of relative entropy. Our results for the convergence rate in terms of relative entropy complement the conventional ones in the strong and weak sense and induce some other properties of the Euler–Maruyama scheme. For example, the convergence in terms of the total variation distance can be implied by Pinsker’s inequality directly. Moreover, when the drift is β(0<β<1)-Hölder continuous in the spatial variable, the convergence rate in terms of the weighted variation distance is also established. Both of these convergence results do not seem to be directly obtained from any other convergence results of the Euler–Maruyama scheme. The main tool this paper relies on is the Girsanov transform.

## 1. Introduction

Consider the following *d*-dimensional stochastic differential equation (SDE)

(1) $$dX_{t}=b\left(X_{t}\right)dt+\sigma \left(X_{t}\right)dW_{t},\;\;\;t\geq 0,\;\;X_{0}=x\in R^{d},$$

where $b:R^{d}\to R^{d}$, $\sigma :R^{d}\to R^{d}⊗R^{m}$, and ${\left(W_{t}\right)}_{t\geq 0}$ is *m*-dimensional Brownian motion on some complete filtration probability space $\left(\Omega ,F,{\left(F_{t}\right)}_{t\geq 0},P\right)$.

Usually, it can be proved that (1) has strong well-posedness under reasonable conditions, whereas the explicit representation of (1) is unknown. Instead, one may develop various numerical schemes to approximate (1); see [1] and references therein for more introductions. When the coefficients are regular, strong/weak convergence of numerical schemes for SDEs have been investigated considerably; see, for instance, monographs [1].

One of the most popular numerical schemes for SDEs is the Euler–Maruyama (EM) scheme, the introduction of which can be found in [1] and references therein. The Euler–Maruyama scheme is a numerical method commonly used for approximating the solutions of SDEs. SDEs are differential equations that involve both deterministic and stochastic (random) components. They are applied in various fields, including physics, finance, biology, and more. The Euler–Maruyama method is particularly useful for solving SDEs because it is a simple and computationally efficient approach. It is an extension of the Euler method, which is used for solving ordinary differential equations. The Euler–Maruyama method is adapted to handle the stochastic part of the equations. Besides its fundamental tools for the numerical solutions of SDEs, researchers also pay much attention to its convergence rate for SDEs. There are some related works in the literature.

For strong convergence of the EM scheme for SDEs, there are some basic results under irregular coefficients. Yan (2002) [2] uses Meyer–Tanaka’s formula and estimates local times to derive a strong convergence rate for EM schemes to one-dimensional SDEs, for which the drift is Lipschitz continuous and the diffusion is Hölder continuous. Gyöngy and Rásonyi (2011) [3] adopt a Yamada–Watanabe approximation approach to derive strong convergence for EM schemes of one-dimensional SDEs with Hölder-continuous diffusions, but the drifts cannot be Lipschitz continuous. Halidias and Kloeden (2008) [4] established strong convergence for an EM scheme of SDEs with monotone drift, which may be discontinuous. Leobacher and Szölgyenyi (2016) and Müller-Gronbach and Yaroslavtseva (2020) [5,6] investigated strong convergence of the EM scheme for one-dimensional SDEs with piecewise Lipschitz-continuous drifts.

Moreover, there are also some other authors who use some transformation tools to get strong convergence of an EM scheme for more complex cases. Leobacher and Szölgyenyi (2017) [7] studied an EM scheme for the multi-dimensional case, which was extended by Leobacher and Szölgyenyi (2018) [8] to the multi-dimensional and degenerate case. The proofs are based on a transformation that changes the piecewise Lipschitz-continuous drifts into globally Lipschitz-continuous ones. Besides the transformation mentioned above, the Zvonkin transform is an alternative tool to deal with the convergence for an EM scheme of SDEs with irregular coefficients. Bao, Huang and Yuan (2019) and Pamen and Taguchi (2017) [9,10] studied SDEs with Hölder- or Hölder–Dini-continuous drifts. Bao, Huang and Zhang (2022) [11] focused on the integrability condition; see the references in [9,10,11] for more results.

For weak convergence, one can refer to [12,13], wherein the drift satisfies the integrable condition and the main tool is the Girsanov transform.

We should remark that all of the references mentioned above study weak or strong convergence of the EM scheme. As far as we know, there are no results on the convergence in the sense of relative entropy.

In this paper, we further characterize the asymptotic behaviors of an EM scheme for SDEs by studying the convergence rate in terms of the so-called relative entropy. Our main results show that the distribution of the EM iteration of SDEs driven by additive Gaussian noise converges to that of real stochastic process of SDEs in terms of relative entropy. And hence, we can get the asymptotic behaviors of its corresponding EM scheme.

Indeed, while relative entropy is commonly perceived as a measure of the dissimilarity between two probability distributions, it falls short of being considered a metric. This is primarily attributed to its asymmetry concerning the order of its parameters and its inability to satisfy the triangle inequality. Despite not meeting the criteria of a metric, relative entropy maintains strong connections with various other metrics. Some notable relationships include: total variation distance, Fisher information divergence, Wasserstein distance and so on; see, e.g., [14] and references therein. These relationships highlight the versatility of relative entropy and its role in connecting with various other measures of dissimilarity and divergence between probability distributions. While it may not possess all the properties of a metric, its specific characteristics make it a valuable tool in information theory and related fields.

Relative entropy’s broad utilization across a spectrum of disciplines, spanning probability theory, statistics, statistical physics, machine learning, neural science, and information theory, can be attributed to its possession of numerous advantageous properties; see, e.g., [15] and references therein. These qualities make it a versatile and valuable tool in diverse applications and fields of study. In addition to conventional convergence analysis in both the strong and weak senses for EM schemes, the relative entropy convergence in our findings could unveil previously unexplored facets of SDEs. These discoveries hold the potential to reveal new properties of SDEs in uncharted research territories, presenting exciting prospects for further exploration and investigation.

As an example of the direct corollary of our main results, the convergence in total variation of an EM scheme can be implied by the well-known Pinsker’s inequality. Pinsker’s inequality states that the relative entropy between two probability measures provides an upper bound for their total variation distance.

Moreover, when the drift of SDE (4) is β(0<β<1)-Hölder-continuous in the spatial variable, the convergence rate for the weighted variation distance will also be induced.

The paper is organized as follows: In Section 2, we review some related concepts and definitions of this paper. In Section 3, we state our assumptions and introduce the main results. In Section 4, we induce the proofs of all results. And conclusions and discussions are provided in Section 5.

## 2. Preliminaries

In this section, we first state some definitions and some related concepts for the main results of this paper.

### 2.1. Euler–Maruyama Scheme

The Euler–Maruyama scheme is a numerical method commonly used for approximating solutions to SDEs. SDEs involve both deterministic and stochastic components, making their solutions more challenging than those of ordinary differential equations. The basic idea is similar to the traditional Euler method for ordinary differential equations but adapted to handle the stochastic terms. The Euler–Maruyama scheme is one of the simplest time-discrete approximations of Itô’s process, and it is sometimes called the Euler–Maruyama aprproximation or Euler approximation.

We consider an Itô’s process satisfying the stochastic differential Equation (1):

$$dX_{t}=b\left(X_{t}\right)dt+\sigma \left(X_{t}\right)dW_{t},\;\;\;t\geq 0,\;\;X_{0}=x\in R^{d},$$

on time interval [0,T] with initial value *x*.

For a given discretization $0=\tau_{0}<\tau_{1}<\cdots <\tau_{N}=T$ of the time interval [0,T], a discrete EM scheme satisfies the following iterative scheme:

(2) $$Y_{n+1}=Y_{n}+b\left(Y_{n}\right)\left(\tau_{n+1}-\tau_{n}\right)+\sigma \left(Y_{n}\right)\left(W_{\tau_{n+1}}-W_{\tau_{n}}\right),$$

for n=0,1,2,⋯,N−1 with initial value $Y_{0}=X_{0}.$ We shall also write $\Delta_{n}=\tau_{n+1}-\tau_{n}$ for the *n*th time increment and call $\delta =\max_{n}\Delta_{n}$ the maximum time step.

In this paper, we shall consider equidistant discretizaiton times $\tau_{n}=n\delta$ with δ=T/N for some integer *N* large enough so that δ∈(0,1). When the diffusion coefficient is identically zero—that is, when σ=0—the stochastic iterative scheme reduces to the deterministic Euler scheme for the ordinary differential equation $dX_{t}=b\left(X_{t}\right)dt$. The main difference is that we need to generate the random increments $\Delta W_{n}=W_{\tau_{n+1}}-W_{\tau_{n}}$ for n=0,1,⋯,N−1 of the Wiener process $W=\{W_{t},t\geq 0\}$. From the properties of Wiener processes, we know that these increments are independent Gaussian random variables with mean $E(\Delta W_{n})=0$ and variance $E\left({\left(\Delta W_{n}\right)}^{2}\right)=\Delta_{n}$. We can use a sequence of independent Gausssian pseudo-random numbers generated by one of the random number generators for the increments of the Wiener process.

The recursive structure of the discrete EM scheme, which evaluates approximate values for the Itô’s process at the discretization instants only, is the key to its successful implementation for numerical approximation of SDEs. For a given time discretizaiton, the discrete EM scheme determines values of the approximation process at the discretization times only. We also need the continuous EM scheme, i.e.,

$$dX_{t}^{\delta }=b\left(X_{t_{\delta }}^{\delta }\right)dt+\sigma \left(X_{t_{\delta }}^{\delta }\right)dW_{t},\;\;\;t\geq 0,\;\;X_{0}^{\delta }=x\in R^{d},$$

where $t_{\delta }:=\left\lfloor t/\delta \right\rfloor \delta$, and ⌊t/δ⌋ is the integer part of t/δ. More details and introductions can be found in [1] and references therein.

### 2.2. Relative Entropy

Kullback and Leibler (1951) [16] firstly introduced the definition of relative entropy, which is also called Kullback–Leibler divergence (K-L divergence for short). The definition of relative entropy is as bellow.

**Definition** **1** (Relative entropy)**.** *Recall that the relative entropy of two probability measures ν and μ on $R^{d}$ is defined as*

$$\mathrm{Ent}\left(\nu |\mu \right)=\left\{\begin{array}{cc} \int_{R^{d}}\log\left(\frac{d\nu }{d\mu }\right)d\nu , & \nu \ll \mu ;\\ \infty , & \mathrm{otherwise}, \end{array}\right.$$

*where $\frac{d\nu }{d\mu }$ is the Radon–Nikodym derivative of ν with respect to μ.*

Relative entropy is a concept from information theory and probability theory that measures how one probability distribution diverges from another one. Roughly speaking, the relative entropy between two probability measures is a measure of the “distance” or difference measuring how “close” these two probability distributions are. In Chapter 4 of reference [17], the authors provide lots of properties of relative entropy (K-L divergence), establish the relationship between relative entropy, cross entropy and conventional differential entropy and give some examples of relative entropy calculations: for instance, exponential distributions, normal distributions and Poisson distributions.

### 2.3. Total Variation and Weighted Variation Distance

**Definition** **2** (Total variation)**.** *For two probability measures $\gamma ,\tilde{\gamma }$ on $R^{d}$, the total variation distance is formulated as*

$$∥\gamma -\tilde{\gamma }{∥}_{var}=\underset{{∥f∥}_{\infty }\leq 1}{\sup}\left|\gamma \left(f\right)-\tilde{\gamma }\left(f\right)\right|.$$

**Definition** **3** (Pinsker’s inequality)**.** 

(3) $$\begin{array}{c} ∥\gamma -\tilde{\gamma }{∥}_{var}^{2}\leq 2\mathrm{Ent}\left(\gamma |\tilde{\gamma }\right). \end{array}$$

**Remark** **1.** *In view of (3), the convergence of the total variation distance can be implied by Pinsker’s inequality directly under the relative entropy convergence.*

**Definition** **4** (Weighted variation distance)**.** *For any k>1, the weighted variation distance for two probability measures $\gamma ,\tilde{\gamma }$ on $R^{d}$ is formulated as*

$$∥\gamma -\tilde{\gamma }{∥}_{k,var}=\underset{{\left|f|<1+|\cdot \right|}^{k}}{\sup}\left|\gamma \left(f\right)-\tilde{\gamma }\left(f\right)\right|.$$

**Remark** **2.** *The convergence of the weighted total variation distance cannot be implied by the relative entropy convergence, so we need to further investigate it.*

### 2.4. Stochastic Differential Equation Description

**Definition** **5** (Stochastic differential equations with additive noise)**.** *In this paper, we consider the following SDE*

(4) $$dX_{t}=b\left(X_{t}\right)dt+dW_{t},\;\;\;t\geq 0,\;\;X_{0}=x\in R^{d},$$

*where $b:R^{d}\to R^{d}$, and ${\left(W_{t}\right)}_{t\geq 0}$ is d-dimensional Brownian motion on some complete filtration probability space $\left(\Omega ,F,{\left(F_{t}\right)}_{t\geq 0},P\right)$.*

**Remark** **3.** *For any δ∈(0,1), the continuous Euler–Maruyama (EM) method for (4) is defined as*

(5) $$dX_{t}^{\delta }=b\left(X_{t_{\delta }}^{\delta }\right)dt+dW_{t},\;\;\;t\geq 0,\;\;X_{0}^{\delta }=x\in R^{d},$$

*with $t_{\delta }:=\left\lfloor t/\delta \right\rfloor \delta$, where ⌊t/δ⌋ is the integer part of t/δ.*

## 3. Assumptions and Main Results

### 3.1. Assumptions

Throughout the paper, we impose the following assumptions on the drift term *b* of the SDE (4).

(**A**) There exists a constant β∈(0,1] and K>0 such that
  (6) $$\begin{array}{c} \left|b\left(x\right)-b\left(y\right)\right|\leq {K|x-y|}^{\beta },\;\;\;x,y\in R^{d} \end{array}$$

**Remark** **4.** *By Zvonkin’s transform introduced in [18], under assumption (A), (4) has a unique strong solution ${\left(X_{t}\right)}_{t\geq 0}$ (see, for instance, [19]).*

### 3.2. Main Results

Let $L_{\xi }$ denote the distribution of a random variable ξ. The main result is the following theorem.

**Theorem** **1.** *Assume the dirft term of the SDE (4) satisfies assumption (* **A** *). Then there exists constants $C_{T,x,d}$ such that*

(7) $$\mathrm{Ent}\left(L_{X_{t}^{\delta }}|L_{X_{t}}\right)\leq KC_{T,x,d}t\delta^{\beta },\;\;t\in \left[0,T\right].$$

*Consequently, we have*

$$\lim_{\delta \to 0}\mathrm{Ent}\left(L_{X_{t}^{\delta }}|L_{X_{t}}\right)=0.$$

**Remark** **5.** *Theorem 1 gives the convergence rate of an EM scheme in the sense of relative entropy for SDEs (4) with additive noise, so its asymptocic behaviors can be established. The main tool of the proof relies on the Girsanov transform. The details of the proof can be found in Section 4.*

**Corollary** **1.** *When assumption (* **A** *) is satisfied, we have*

$$∥L_{X_{t}^{\delta }}-L_{X_{t}}{∥}_{var}\leq \sqrt{2KC_{T,x,d}t}\delta^{\frac{\beta }{2}},\;\;t\in \left[0,T\right].$$

**Remark** **6.** *Corollary 1 is the convergence of an EM scheme for the total variance distance. This can be implied by Pinker’s inequality (3) directly.*

**Theorem** **2.** *If assumption (* **A** *) holds for some 0<β<1, then for any k≥1 there exists a constant $c_{k,T,x,d}$ such that*

$$∥L_{X_{t}^{\delta }}\left(f\right)-L_{X_{t}}{\left(f\right)∥}_{k,var}=\underset{{\left|f|<1+|\cdot \right|}^{k}}{\sup}\left|L_{X_{t}^{\delta }}\left(f\right)-L_{X_{t}}\left(f\right)\right|\leq c_{k,T,x,d}\sqrt{t}\delta^{\frac{\beta }{2}},\;\;t\in \left[0,T\right].$$

**Remark** **7.** *Theorem 2 is the convergence of an EM scheme for the weighted total variance distance. This convergence is not the direct application of the convergence in the relative entropy sense for the EM scheme of Theorem 1. And the details of the proof can be found in Section 4.*

## 4. Proofs

### 4.1. Proof of Theorem 1

Before finishing the proof of Theorem 1, we prepare some auxiliary lemmas. The first lemma below plays a crucial role in the proof of Theorem 1.

**Lemma** **1.** *Assume* **(A)***. Then for any k≥1, there exists a constant $C_{T,d,k}>0$ such that*

(8) $$E\underset{t\in [0,T]}{\sup}|X_{t}^{\delta }{|}^{k}+E\underset{t\in [0,T]}{\sup}|X_{t}{|}^{k}\leq C_{T,d,k}{\left(1+|x\right|}^{k}),\;\;t\in \left[0,T\right].$$

**Proof.**  Without loss of generality, we only prove the inequality for $X_{t}^{\delta }$ since it is similar for $X_{t}$.

For any n≥1, let $\zeta_{n}:=\inf\{t\geq 0,|X_{t}^{\delta }\left|\geq n\right\}$. Firstly, we have

(9) $$\begin{array}{c} |X_{t\wedge \zeta_{n}}^{\delta }{|}^{k}\leq 2^{k-1}{\left|\int_{0}^{t\wedge \zeta_{n}}b\left(X_{s_{\delta }}^{\delta }\right)ds\right|}^{k}+2^{k-1}{\left|W_{t\wedge \zeta_{n}}\right|}^{k},\;\;t\in \left[0,T\right]. \end{array}$$

By **(A)**, it is easy to see that

(10) $$\begin{array}{c} \left|b\left(x\right)\right|\leq \left|b\left(x\right)-b\left(0\right)|+|b\left(0\right)\right|\leq {K|x|}^{\beta }+|b\left(0\right)|\leq K(1+|x|)+|b\left(0\right)|,\;\;x\in R^{d}. \end{array}$$

Combining this with (9), we can find a constant $c_{0}>0$ such that

(11) $$\begin{array}{c} E\underset{t\in [0,r]}{\sup}|X_{t\wedge \zeta_{n}}^{\delta }{|}^{k}\leq c_{0}TE\int_{0}^{r}(1+\underset{t\in [0,s]}{\sup}|X_{t\wedge \zeta_{n}}^{\delta }{|}^{k})ds+2^{k-1}E\underset{t\in [0,r]}{\sup}{\left|W_{t\wedge \zeta_{n}}\right|}^{k},\;\;r\in \left[0,T\right]. \end{array}$$

By the Burkerholder–Davis–Gundy inequality, there exists a constant $c_{1}>0$ such that

$$E\underset{t\in [0,r]}{\sup}{\left|W_{t\wedge \zeta_{n}}\right|}^{k}\leq c_{1}{\left(dr\right)}^{\frac{k}{2}},\;\;r\in \left[0,T\right].$$

Putting this into (11) and applying Gronwall’s inequality, we find a constant $C_{T}>0$ such that

$$E\underset{t\in [0,T]}{\sup}|X_{t\wedge \zeta_{n}}^{\delta }{|}^{k}\leq C_{T,d,k}{\left(1+|x\right|}^{k}).$$

Note that

$$P\left(\zeta_{n}<T\right)\leq P(|X_{T\wedge \zeta_{n}}^{\delta }\left|\geq n\right)\leq \frac{E\sup_{t\in [0,T]}{\left|X_{t\wedge \zeta_{n}}^{\delta }\right|}^{k}}{n^{k}}\leq \frac{C_{T,d,k}{\left(1+|x\right|}^{k})}{n^{k}}.$$

This yields that P-a.s. $\lim_{n\to \infty }\zeta_{n}=\infty$, which, combined with Fatou’s lemma, yields that

$$E\underset{t\in [0,T]}{\sup}|X_{t}^{\delta }{|}^{k}\leq \underset{n\to \infty }{lim inf}E\underset{t\in [0,T]}{\sup}|X_{t\wedge \zeta_{n}}^{\delta }{|}^{k}\leq C_{T,d,k}{\left(1+|x\right|}^{k}).$$

So we complete the proof. □

**Lemma** **2.** *Under (A), there exists a constant $C_{T,x,d}>0$ such that*

(12) $$\underset{t\in [0,T]}{\sup}E{\left|X_{t}^{\delta }-X_{t_{\delta }}^{\delta }\right|}^{4}\leq C_{T,x,d}\delta .$$

**Proof.**  Note that

$$X_{t}^{\delta }-X_{t_{\delta }}^{\delta }=\int_{t_{\delta }}^{t}b\left(X_{s_{\delta }}^{\delta }\right)ds+W_{t}-W_{t_{\delta }}$$

This together with (10), Lemma 1 and the fact $E|W_{t}-W_{t_{\delta }}{|}^{4}=\left(d^{2}+2d\right){\left(t-t_{\delta }\right)}^{2}\leq \left(d^{2}+2d\right)\delta^{2}$ implies that

$$\begin{array}{cc} E|X_{t}^{\delta }-X_{t_{\delta }}^{\delta }{|}^{4} & \leq 8\delta^{3}\int_{t_{\delta }}^{t}E{\left|b\left(X_{s_{\delta }}^{\delta }\right)\right|}^{4}ds+8\left(d^{2}+2d\right)\delta^{2}\\ \; & \leq 8\delta^{3}C_{0}\int_{t_{\delta }}^{t}E(1+\underset{t\in [0,T]}{\sup}|X_{t}^{\delta }{|}^{4})ds+8\left(d^{2}+2d\right)\delta^{2}\\ \; & \leq C_{T,x,d}\delta^{2}. \end{array}$$

Therefore, the proof is completed. □

**Lemma** **3.** *Assume* **(A)** *. Then there exist constants $\kappa_{1}\left(T\right),\kappa_{2}\left(T,x,d\right)>0$ such that*

$$E\exp\left\{\kappa_{1}\left(T\right)\underset{t\in [0,r]}{\sup}{\left|X_{t}\right|}^{2}\right\}\leq \kappa_{2}\left(T,x,d\right),\;\;r\in \left[0,T\right].$$

**Proof.**  By Itô’s formula, we can find a constant $c_{0}>0$ such that

$$\begin{array}{cc} |X_{t}{|}^{2} & \leq {\left|x\right|}^{2}+\int_{0}^{t}\langle b\left(X_{s}\right),2X_{s}\rangle ds+\int_{0}^{t}\langle 2X_{s},dW_{s}\rangle +dt\\ \; & \leq {\left|x\right|}^{2}+\left(c_{0}+d\right)t+c_{0}\int_{0}^{t}{\left|X_{s}\right|}^{2}ds+\int_{0}^{t}\langle 2X_{s},dW_{s}\rangle . \end{array}$$

Gronwall’s inequality yields that

(13) $$\begin{array}{c} \underset{t\in [0,r]}{\sup}{\left|X_{t}\right|}^{2}\leq e^{c_{0}r}\left[{\left|x\right|}^{2}+\left(c_{0}+d\right)r+\underset{t\in [0,r]}{\sup}\int_{0}^{t}\langle 2X_{s},dW_{s}\rangle \right],\;\;r\in \left[0,T\right]. \end{array}$$

Note that for any ε>0,

(14) $$\begin{array}{cc} E\exp\left\{\varepsilon \underset{t\in [0,r]}{\sup}\int_{0}^{t}\langle 2X_{s},dW_{s}\rangle \right\} & \leq eE\exp\left\{\varepsilon \int_{0}^{r}\langle 2X_{s},dW_{s}\rangle \right\}\\ \; & \leq e{\left\{Ee^{8\varepsilon^{2}\int_{0}^{r}{\left|X_{s}\right|}^{2}ds}\right\}}^{\frac{1}{2}}\\ & \leq e{\left\{Ee^{8\varepsilon^{2}r\;\sup_{s\in [0,r]}{\left|X_{s}\right|}^{2}}\right\}}^{\frac{1}{2}},\;\;r\in \left[0,T\right]. \end{array}$$

Taking $\varepsilon =\frac{1}{8T}e^{-c_{0}T}$, we have $\varepsilon e^{-c_{0}T}=8T\varepsilon^{2}$. Combining this with (13) and (14), we derive

$$\begin{array}{cc} E\exp\left\{\varepsilon e^{-c_{0}T}\underset{t\in [0,T]}{\sup}{\left|X_{t}\right|}^{2}\right\} & \leq {\exp\{\varepsilon [|x|}^{2}+\left(c_{0}+d\right)T]\}E\exp\left\{\varepsilon \underset{t\in [0,T]}{\sup}\int_{0}^{t}\langle 2X_{s},dW_{s}\rangle \right\}\\ \; & \leq {\exp\{\varepsilon [|x|}^{2}+\left(c_{0}+d\right)T]\}e{\left\{Ee^{8\varepsilon^{2}T\;\sup_{s\in [0,T]}{\left|X_{s}\right|}^{2}}\right\}}^{\frac{1}{2}}. \end{array}$$

By a stopping time technique, we may and do assume that

$$E\exp\left\{\varepsilon e^{-c_{0}T}\underset{t\in [0,T]}{\sup}{\left|X_{t}\right|}^{2}\right\}<\infty .$$

Then we get

$$E\exp\left\{\frac{1}{8T}e^{-2c_{0}T}\underset{t\in [0,T]}{\sup}{\left|X_{t}\right|}^{2}\right\}\leq \exp\left\{2+\frac{1}{4T}e^{-c_{0}T}{\left[|x\right|}^{2}+\left(c_{0}+d\right)T]\right\}.$$

Letting $\kappa_{1}\left(T\right)=\frac{1}{8T}e^{-2c_{0}T}$ and $\kappa_{2}\left(T,x,d\right)=\exp\left\{2+\frac{1}{4T}e^{-c_{0}T}{\left[|x\right|}^{2}+\left(c_{0}+d\right)T]\right\}$, we complete the proof. □

**Proof of Theorem** **1.** For any n≥1, let $\tau_{n}:=\inf\{t\geq 0,|X_{t}\left|\vee \right|X_{t}^{\delta }\left|\geq n\right\}$. By Lemma 1, it holds that P-a.s. $\lim_{n\to \infty }\tau_{n}=\infty$. Recall that

(15) $$\begin{array}{c} dX_{t}=b\left(X_{t}\right)dt+dW_{t},\;\;t\in \left[0,T\right]. \end{array}$$

Let

(16) $$\begin{array}{c} W_{t}^{\delta }=W_{t}-\int_{0}^{t}\left[b\left(X_{s_{\delta }}\right)-b\left(X_{s}\right)\right]ds,\;\;t\in \left[0,T\right]. \end{array}$$

Then (15) can be rewritten as

$$dX_{t}=b\left(X_{t_{\delta }}\right)dt+dW_{t}^{\delta },\;\;t\in \left[0,T\right].$$

Set

$$R_{t}=\exp\left\{\int_{0}^{t}\langle \left[b\left(X_{s_{\delta }}\right)-b\left(X_{s}\right)\right],dW_{s}\rangle -\frac{1}{2}\int_{0}^{t}{\left|b\left(X_{s_{\delta }}\right)-b\left(X_{s}\right)\right|}^{2}ds\right\},\;\;t\in \left[0,T\right].$$

Fix $t_{0}\in \left(0,T\right]$. By **(A)** and Girsanov’s theorem, we conclude that ${\left\{R_{t\wedge \tau_{n}}\right\}}_{t\in [0,t_{0}]}$ is a martingale and $W_{t}^{\delta }$ is *d*-dimensional Brownian motion up to $t_{0}\wedge \tau_{n}$ under probability measure $Q^{n}=R_{t_{0}\wedge \tau_{n}}P$. This together with **(A)** and Lemma 2 implies that

(17) $$\begin{array}{cc} & E^{Q^{n}}\int_{0}^{t_{0}\wedge \tau_{n}}{\left|b\left(X_{s_{\delta }}\right)-b\left(X_{s}\right)\right|}^{2}ds\\ \; & =E\int_{0}^{t_{0}\wedge \tau_{n}}{\left|b\left(X_{s_{\delta }}^{\delta }\right)-b\left(X_{s}^{\delta }\right)\right|}^{2}ds\\ & \leq E\int_{0}^{t_{0}}K^{2}{\left|X_{s_{\delta }}^{\delta }-X_{s}^{\delta }\right|}^{2\beta }ds\\ & \leq \int_{0}^{t_{0}}K^{2}\left\{E\right|X_{s_{\delta }}^{\delta }-X_{s}^{\delta }{{|}^{2}\}}^{\beta }ds\leq K^{2}C_{T,x,d}t_{0}\delta^{\beta }. \end{array}$$

From this and (16), we derive that

$$\begin{array}{cc} E[R_{t_{0}\wedge \tau_{n}}\log R_{t_{0}\wedge \tau_{n}}] & =E^{Q^{n}}\left[\log R_{t_{0}\wedge \tau_{n}}\right]\\ \; & =E^{Q^{n}}\int_{0}^{t_{0}\wedge \tau_{n}}\langle \left[b\left(X_{s_{\delta }}\right)-b\left(X_{s}\right)\right],dW_{s}\rangle -\frac{1}{2}E^{Q^{n}}\int_{0}^{t_{0}\wedge \tau_{n}}{\left|b\left(X_{s_{\delta }}\right)-b\left(X_{s}\right)\right|}^{2}ds\\ \; & =E^{Q^{n}}\int_{0}^{t_{0}\wedge \tau_{n}}\langle \left[b\left(X_{s_{\delta }}\right)-b\left(X_{s}\right)\right],dW_{s}^{\delta }\rangle +\frac{1}{2}E^{Q^{n}}\int_{0}^{t_{0}\wedge \tau_{n}}{\left|b\left(X_{s_{\delta }}\right)-b\left(X_{s}\right)\right|}^{2}ds\\ \; & =\frac{1}{2}E^{Q^{n}}\int_{0}^{t_{0}\wedge \tau_{n}}{\left|b\left(X_{s_{\delta }}\right)-b\left(X_{s}\right)\right|}^{2}ds\\ \; & \leq \frac{1}{2}K^{2}C_{T,x,d}t_{0}\delta^{\beta }. \end{array}$$

This combined with the convergence theorem of martingales implies that ${\left\{R_{t}\right\}}_{t\in [0,t_{0}]}$ is a martingale, and it follows from Fatou’s lemma that

$$\begin{array}{cc} E\left[R_{t_{0}}\log R_{t_{0}}\right]\leq \lim_{n\to \infty }E\left[R_{t_{0}\wedge \tau_{n}}\log R_{t_{0}\wedge \tau_{n}}\right]\; & \leq \frac{1}{2}K^{2}C_{T,x,d}t_{0}\delta^{\beta }. \end{array}$$

Applying Girsanov’s theorem again, we conclude that ${\left\{R_{t}\right\}}_{t\in [0,t_{0}]}$ is a martingale and $W_{t}^{\delta }$ is *d*-dimensional Brownian motion up to $t_{0}$ under probability measure $Q=R_{t_{0}}P$, and hence, the distribution of ${\left\{X_{t}\right\}}_{t\in [0,t_{0}]}$ under Q is equal to that of ${\left\{X_{t}^{\delta }\right\}}_{t\in [0,t_{0}]}$ under P. As a result, it holds that

(18) $$\begin{array}{c} Ef\left(X_{t_{0}}^{\delta }\right)=E^{Q}f\left(X_{t_{0}}\right)=E\left[R_{t_{0}}f\left(X_{t_{0}}\right)\right],\;\;|f|\leq {1+|\cdot |}^{k},k\geq 1. \end{array}$$

By Young’s inequality, we derive that

(19) $$\mathrm{Ent}\left(L_{X_{t_{0}}^{\delta }}|L_{X_{t_{0}}}\right)\leq C_{T,x,d}\delta^{\beta },\;\;t\in \left[0,T\right].$$

The proof is completed. □

### 4.2. Proof of Corollary 1

Corollary 1 is the direct result of Theorem 1 and Pinsker’s inequality (3).

### 4.3. Proof of Theorem 2

**Proof.**  Firstly, we have

$$\begin{array}{cc} & E{\left[R_{t_{0}}-1\right]}^{2}=E\left[R_{t_{0}}^{2}\right]-1\\ \; & =E\{\exp\left\{2\int_{0}^{t_{0}}\langle \left[b\left(X_{s_{\delta }}\right)-b\left(X_{s}\right)\right],dW_{s}\rangle -4\int_{0}^{t_{0}}{\left|b\left(X_{s_{\delta }}\right)-b\left(X_{s}\right)\right|}^{2}ds\right\}\\ \; & \;\;\times \exp\left\{3\int_{0}^{t_{0}}{\left|b\left(X_{s_{\delta }}\right)-b\left(X_{s}\right)\right|}^{2}ds\right\}\}-1\\ \; & \leq {\left\{E\exp\left\{6\int_{0}^{t_{0}}{\left|b\left(X_{s_{\delta }}\right)-b\left(X_{s}\right)\right|}^{2}ds\right\}\right\}}^{\frac{1}{2}}-1\\ \; & \leq E\exp\left\{6\int_{0}^{t_{0}}{\left|b\left(X_{s_{\delta }}\right)-b\left(X_{s}\right)\right|}^{2}ds\right\}-1\\ \; & \leq E\left[\exp\left\{6\int_{0}^{t_{0}}{\left|b\left(X_{s_{\delta }}\right)-b\left(X_{s}\right)\right|}^{2}ds\right\}6\int_{0}^{t_{0}}{\left|b\left(X_{s_{\delta }}\right)-b\left(X_{s}\right)\right|}^{2}ds\right]\\ \; & \leq 6{\left\{E\exp\left\{12\int_{0}^{t_{0}}{\left|b\left(X_{s_{\delta }}\right)-b\left(X_{s}\right)\right|}^{2}ds\right\}\right\}}^{\frac{1}{2}}{\left[E{\left(\int_{0}^{t_{0}}{\left|b\left(X_{s_{\delta }}\right)-b\left(X_{s}\right)\right|}^{2}ds\right)}^{2}\right]}^{\frac{1}{2}}\\ \; & \leq 6{\left\{E\exp\left\{48K^{2}t_{0}\underset{s\in [0,t_{0}]}{\sup}{\left|X_{s}\right|}^{2\beta }ds\right\}\right\}}^{\frac{1}{2}}{\left[E{\left(\int_{0}^{t_{0}}{\left|b\left(X_{s_{\delta }}\right)-b\left(X_{s}\right)\right|}^{2}ds\right)}^{2}\right]}^{\frac{1}{2}} \end{array}$$

By Lemma 3 and for β<1, we derive

$$6{\left\{E\exp\left\{48K^{2}t_{0}\underset{s\in [0,t_{0}]}{\sup}{\left|X_{s}\right|}^{2\beta }ds\right\}\right\}}^{\frac{1}{2}}<C\left(T,x,d\right)$$

for some constant C(T,x,d)>0. Moreover, Hölder’s inequality and Lemma 2 imply that

$$\begin{array}{cc} \; & {\left[E{\left(\int_{0}^{t_{0}}{\left|b\left(X_{s_{\delta }}\right)-b\left(X_{s}\right)\right|}^{2}ds\right)}^{2}\right]}^{\frac{1}{2}}\leq \sqrt{t_{0}}{\left[\int_{0}^{t_{0}}E{\left|b\left(X_{s_{\delta }}\right)-b\left(X_{s}\right)\right|}^{4}ds\right]}^{\frac{1}{2}}\leq c_{1}t_{0}\delta^{\beta }. \end{array}$$

So we conclude that

$$E{\left[R_{t_{0}}-1\right]}^{2}\leq ct_{0}\delta^{\beta }$$

for some constant c>0. From this together with (18) and Lemma 1, we derive

$$\begin{array}{cc} \; & ∥L_{X_{t}^{\delta }}\left(f\right)-L_{X_{t}}{\left(f\right)∥}_{k,var}\\ \; & =\underset{{\left|f|<1+|\cdot \right|}^{k}}{\sup}\left|L_{X_{t}^{\delta }}\left(f\right)-L_{X_{t}}\left(f\right)\right|\\ \; & =\underset{{\left|f|<1+|\cdot \right|}^{k}}{\sup}\left|E\left[\left(R_{t}-1\right)f\left(X_{t}\right)\right]\right|\\ \; & \leq {\left[E{\left(R_{t}-1\right)}^{2}\right]}^{\frac{1}{2}}\left[E(1+\right|X_{t}{|}^{k}{{)}^{2}]}^{\frac{1}{2}}\\ \; & \leq c_{k,T,x,d}\sqrt{t}\delta^{\frac{\beta }{2}}. \end{array}$$

The proof is completed. □

## 5. Conclusions and Discussion

In this paper, we studied the convergence rate of the relative entropy for a Euler–Maruyama scheme of stochastic differential equations driven by additive Gaussian noise, and we obtained its asymptotic behaviors. Our results for the convergence rate in terms of relative entropy complement the conventional ones in the strong and weak sense and induce some other properties of the Euler–Maruyama scheme. The convergence in terms of total variation distance can be implied by Pinsker’s inequality directly. And the convergence rate in terms of the weighted variation distance is also established.

Finally, we discuss some more complicated processes.

(1) SDEs with multiplicative noise: The SDE becomes

(20) $$\begin{array}{c} dX_{t}=b\left(X_{t}\right)dt+\sigma \left(X_{t}\right)dW_{t},\;\;X_{0}=x \end{array}$$

The EM scheme satisfies

(21) $$\begin{array}{c} dX_{t}^{\delta }=b\left(X_{t_{\delta }}^{\delta }\right)dt+\sigma \left(X_{t_{\delta }}^{\delta }\right)dW_{t},\;\;X_{0}^{\delta }=x. \end{array}$$

When σ is invertible, we can still rewrite (20) as

(22) $$\begin{array}{c} dX_{t}=b\left(X_{t_{\delta }}\right)dt+\sigma \left(X_{t}\right)d{\tilde{W}}_{t} \end{array}$$

with

$$d{\tilde{W}}_{t}=dW_{t}-\sigma^{-1}\left(X_{t}\right)\left(b\left(X_{t_{\delta }}\right)-b\left(X_{t}\right)\right)dt.$$

Different from the additive noise case, (21) and (22) solve different SDEs and, hence, the Girsanov transform is unavailable. We need to develop new approaches to deal with the multiplicative noise case.

(2) Geometric Brownian motion (GBM): In reference [20], the authors investigated time-averaging and nonergodicity for GBM in the presence of drift and with resetting. Although GBM in the presence of drift has explicit representation and follows log-normal distribution, it solves an SDE with linear and multiplicative noise:

$$dX_{t}=\mu X_{t}dt+\sigma X_{t}dW_{t}.$$

The difficulty appearing in the multiplicative noise case will still exist.

(3) Anomalous-diffusion processes: In reference [21], the authors studied the nonergodicity, non-Gaussianity and aging of scaled fractional Brownian motion. We believe that our present method for estimating the relative entropy between EM scheme and the true solution is also available in SDEs driven by additive fractional Brownian motion since the Girsanov transform can also be used, and the Girsanov transform is relatively simple for the case of $H<\frac{1}{2}$.

We will leave these processes mentioned above for a future study.

## Data Availability

The data presented in this study are available on request from the corresponding author.
